# Effects of Quercetin on Proliferation and H_2_O_2_-Induced Apoptosis of Intestinal Porcine Enterocyte Cells

**DOI:** 10.3390/molecules23082012

**Published:** 2018-08-12

**Authors:** Zhigang Chen, Qiaoling Yuan, Guangren Xu, Huiyu Chen, Hongyu Lei, Jianming Su

**Affiliations:** 1 Department of Basic Veterinary Medicine, College of Veterinary Medicine, Hunan Agricultural University, Changsha 410128, Hunan, China; czgresearcher@163.com (Z.C.); yql0501@126.com (Q.Y.); xugr0818@163.com (G.X.); 2 Department of Preventive Veterinary Medicine, College of Veterinary Medicine, Hunan Agricultural University, Changsha 410128, Hunan, China; chenhuiyuhn@163.com (H.Y.); leihy77@hunau.edu.cn (H.L.)

**Keywords:** hydrogen peroxide, IPEC-J2, oxidative stress, apoptosis, mitochondrial membrane potential, pro-proliferation

## Abstract

Weanling stress and toxicosis, which are harmful to the health of pigs’ intestines, are associated with oxidative stress. Quercetin (Que) is a polyphenolic compound that shows good anti-cancer, anti-inflammation and anti-oxidation effects. This study aimed to elaborate whether or not Que promotes IPEC-J2 (intestinal porcine enterocyte cells) proliferation and protects IPEC-J2 from oxidative damage. Thus, we examined the effects of Que on proliferation and H_2_O_2_-induced apoptosis in IPEC-J2. The results showed that Que increased IPEC-J2 viabililty, propelled cells from G1 phase into S phase and down-regulated gene levels of P27 and P21, respectively. Besides, H_2_O_2_-induced cell damage was alleviated by Que after different exposure times, and Que depressed apoptosis rate, reactive oxygen species (ROS) level and percentage of G1 phase cells and elevated the percentage of cells in G2 phase and S phase and mitochondrial membrane potential (Δψm) after IPEC-J2 exposure to H_2_O_2_. Meanwhile, Que reduced the value of Bax/Bcl-2 in H_2_O_2_ exposed cells. In low-degree oxidative damage cells, lipid peroxidation product malondialdehyde (MDA) content and superoxide dismutase (SOD) activity were increased. In turn, Que could reverse the change of MDA content and SOD activity in low-degree damage cells. Nevertheless, catalase (CAT) activity was not changed in IPEC-J2 incubated with Que under low-degree damage conditions. Interestingly, relative expressive levels of the proteins claudin-1 and occludin were not altered under low-degree damage conditions, but Que could improve claudin-1 and occludin levels, slightly. This research indicates that Que can be greatly beneficial for intestinal porcine enterocyte cell proliferation and it protects intestinal porcine enterocyte cells from oxidation-induced apoptosis, and could be used as a potential feed additive for porcine intestinal health against pathogenic factor-induced oxidative damages and apoptosis.

## 1. Introduction

With the continuous spread of large-scale farming, the intestinal health of pigs is challenged, resulting in problems such as weaning stress, mycotoxicosis and endotoxin-induced stress [1,2,3]. These can lead to oxidative damage and inflammatory response in pigs’ intestines, and subsequently destroy the structure of the intestinal tissue, interfere with the intestinal immunity function, eventually reduce the digestion and absorption of nutrients, and reduce the porcine growth performance [4,5,6,7]. In particular, these can result in death of pigs exposed to serious oxidative damage or inflammatory responses. IPEC-J2, a non-cancerous cell line, is a small intestinal epithelial cell of pigs, which is initially separated from the jejunum of newborn piglets, and this cell line generally has the same physiological state as normal intestinal cells [8,9]. At present, the cell line is widely used to explore *in-vitro* toxicity and mechanism of toxicants, utilization of nutrients as well as screening drug function [10,11,12]. Porcine intestinal epithelial cells have a barrier function against harmful substances [13] and the proliferation of intestinal epithelial cells is closely related to the normal development of the intestinal mucosa and villi, and the increase of intestinal villi height is helpful to improve the absorption function of the small intestine, which indirectly reflects the close relationship between proliferation of small intestinal epithelial cells and the absorption function of the small intestine [14,15], so it is beneficial for intestinal health to promote proliferation of small intestinal epithelial cells. Furthermore, moderate cell apoptosis, a normal life phenomenon, belongs to the programmed cell death process that balances metabolism and maintains normal physiological activity, but the aggravation of apoptosis in normal cells will destroy the organism’s structure and interfere with the basic functions of cells [16]. Moreover, the oxidative stress mediated by reactive oxygen species (ROS) can aggravate oxidation reactions, damage the nucleic acids, proteins and lipids in cells, and may increase cell apoptosis, consequently harming health, via breaking the inherent redox balance of cells [17,18]. Importantly, current reports indicate that an anti-nutritional factor soybean agglutinin, the metal element zinc, the mycotoxin zearalenone and weanling stress are all conducive to aggravating apoptosis in porcine intestinal cells [19,20,21,22].

Herbaceous plants play an important role in traditional medicine. With the continuous and in depth development of research techniques, research workers are constantly identifying new natural molecules in herbaceous plants to explore their biological functions and mechanisms, which makes the effects of plant-derived molecules on health care become a research hot spot in recent years. Based on the above, it is of great significance to screen high safety natural product molecules as veterinary drugs or feed additives to protect pigs’ intestines. Quercetin (Que), a common polyphenol compound, widely distributed in fruits and vegetables, possesses good antioxidation, anti-cancer and anti-inflammatory responses [23,24,25]. Intestinal absorption kinetic experiments in mouse intestinal cells in vivo and human intestinal cells in vitro showed that Que had good intestinal absorption efficiency [26,27]. Ben et al. [28] reported that Que could protect the human colon cancer cell line, HCT116, from exposure to dichlorvos, inhibiting apoptosis by regulating the redox system in HCT116. However, there is not much detailed evidence to support whether or not Que promotes the proliferation of porcine small intestinal epithelial cells, and whether or not Que has any inhibitory effect on oxidative damage in porcine intestinal epithelial cells. 

Meanwhile, hydrogen peroxide (H_2_O_2_), as a kind of free radicals, is often applied to establish an oxidative damage model due to its strong oxidation properties [29,30]. Therefore, in this study, we explored the effects of Que on IPEC-J2 proliferation and the interventive effects and possible mechanisms of Que on IPEC-J2 under oxidative damage conditions induced by H_2_O_2_.

## 2. Results

### 2.1. Effects of Que on IPEC-J2 Viability

**Figure 1:**
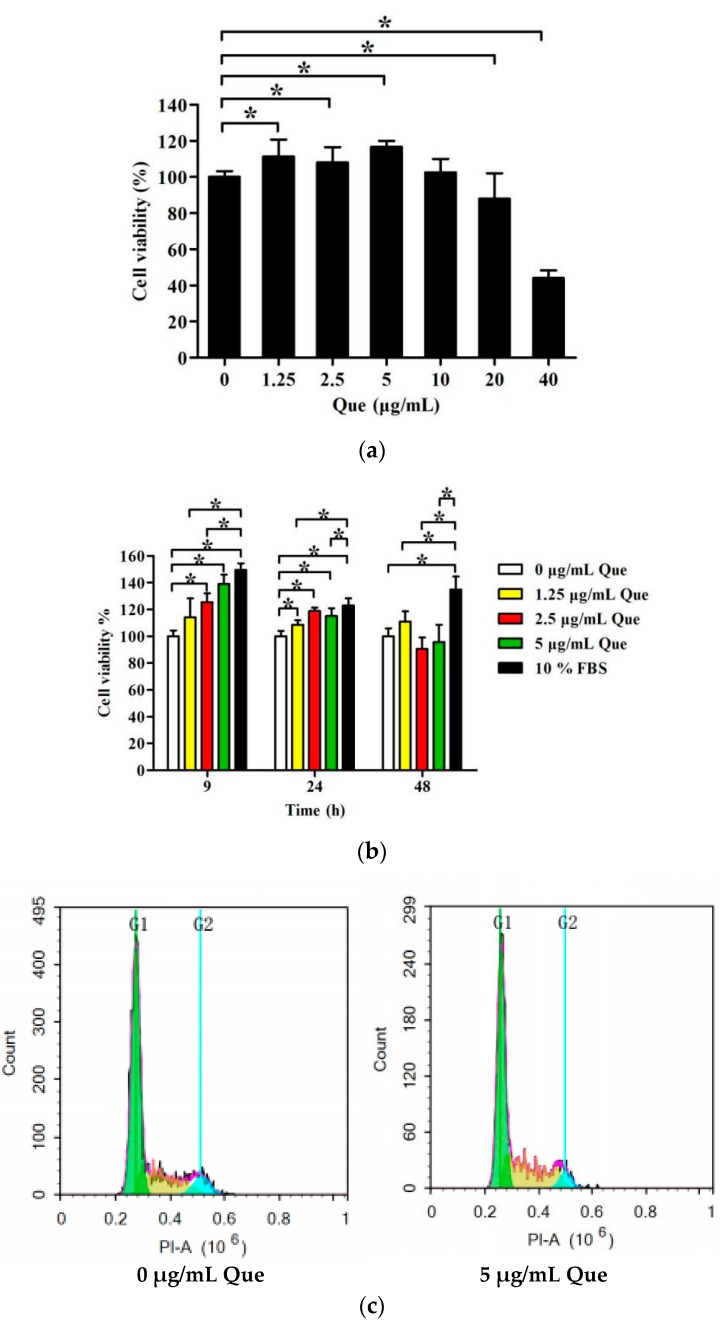

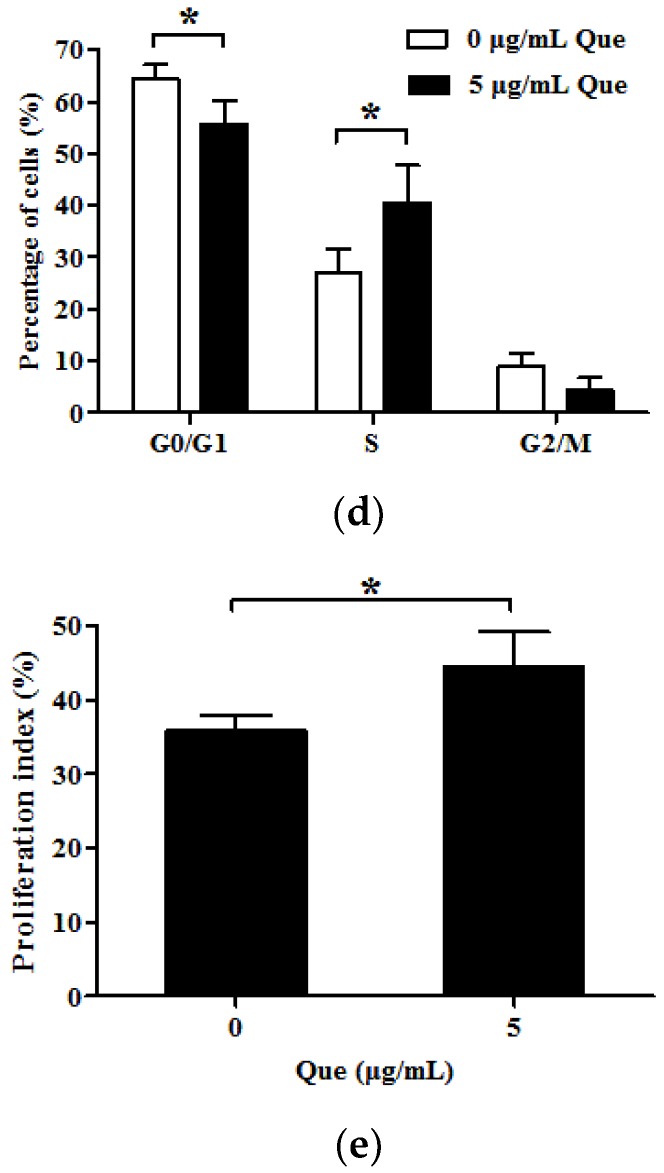
Effects of Que on the viability, cell cycle and proliferation index of IPEC-J2. (**a**) Viability of IPEC-J2 treated with Que (0, 1.25, 2.5, 5, 10, 20 and 40 μg/mL) for 24 h. (**b**) Viability of IPEC-J2 treated with Que (0, 1.25, 2.5, 5 and 10 μg/mL) for 9 h, 24 h, and 48 h. (**c**) Flow detection diagram of cell cycle of IPEC-J2 treated with 0 and 5 μg/mL for 24 h. (**d**) Histogram of cell cycle of IPEC-J2 treated with 0 and 5 μg/mL Que for 24 h. (**e**) Histogram of proliferation index of IPEC-J2 treated with 0 and 5 μg/mL Que for 24 h. Data are presented as mean ± SD. * *p* < 0.05 means significant difference between two groups.

The MTT assay was used to test IPEC-J2 viability after cells were cultured with Que. IPEC-J2 viability was not decreased after treatment with Que at concentrations of no more than 10 μg/mL for 24 h, and Que at concentrations of 1.25, 2.5 and 5 μg/mL remarkably increased cell viability (*p* < 0.05) (Figure 1a). As Figure 1b shows, the pro-proliferative effect of Que was most obvious at 9 h treatment (*p* < 0.05). As time increased, the pro-proliferative effect slowed down. After treatment with 5 μg/mL of Que for 9 h, cell viability was nearly similar to that of the serum-treated group. There was no significant change in viability of IPEC-J2 treated with Que for 48 h, compared with the control group.

### 2.2. Effects of Que on Cell Cycle and Proliferation Index in IPEC-J2

To investigate the effects of Que on cell cycle and proliferation index in IPEC-J2, flow cytometry was applied. Five μg/mL Que treatment for 24 h reduced the proportion of cells in G0/G1 phase and raised the proportion of cells in S phase (*p* < 0.05). However, 5 μg/mL Que did not significantly alter the proportion of G2/M phase cells (*p* > 0.05) (Figure 1c,d). The proliferation index was calculated according to the proportion of the S and G2/M phases to the G0/G1, S and G2/M phases. The proliferation index of IPEC-J2 was increased by 1.24-fold compared to control group after incubation with 5 μg/mL Que, which is basically close to the cell viability results detected via the MTT assay method (*p* < 0.05) (Figure 1e).

### 2.3. Effects of Que on mRNA Relative Expression Level of P21 and P27 of IPEC-J2

**Figure 2:**
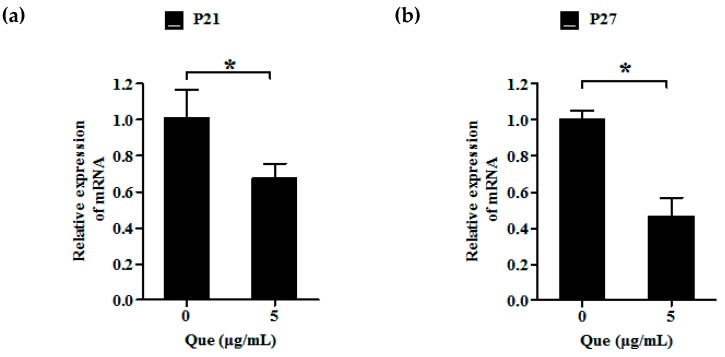
Effects of Que on mRNA relative expression level of P21 and P27 of IPEC-J2. (**a**) Relative expression level of P21 of IPEC-J2. (**b**) Relative expression level of P27 of IPEC-J2. Data are presented as mean ± SD. * *p* < 0.05 means significant difference between two groups.

To further examine the pro-proliferative effect of Que on IPEC-J2, we measured the mRNA relative expression level of P21 and P27 by real-time PCR. Five μg/mL Que treatment dramatically decreased P21 and P27 gene expression levels, respectively (*p* < 0.05) (Figure 2a,b).

### 2.4. Effects of Que on Repair of IPEC-J2 after Scratching

In order to investigate whether Que enhances repair of IPEC-J2 after scratching, the cell damage rate was determined by a manual lineation method. As shown in Figure S1, 1.25, 2.5 and 5 μg/mL Que did not reduce the rate of IPEC-J2 damage after scratching. More importantly, 5 μg/mL Que showed a damage-exacerbating effect on IPEC-J2 under continuous cultured with cells for 72 h (*p* < 0.05).

### 2.5. Effects of H_2_O_2_ on Viability of IPEC-J2

**Figure 3:**
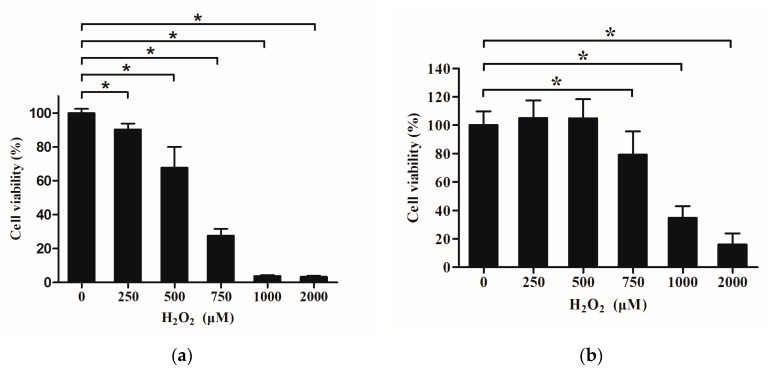

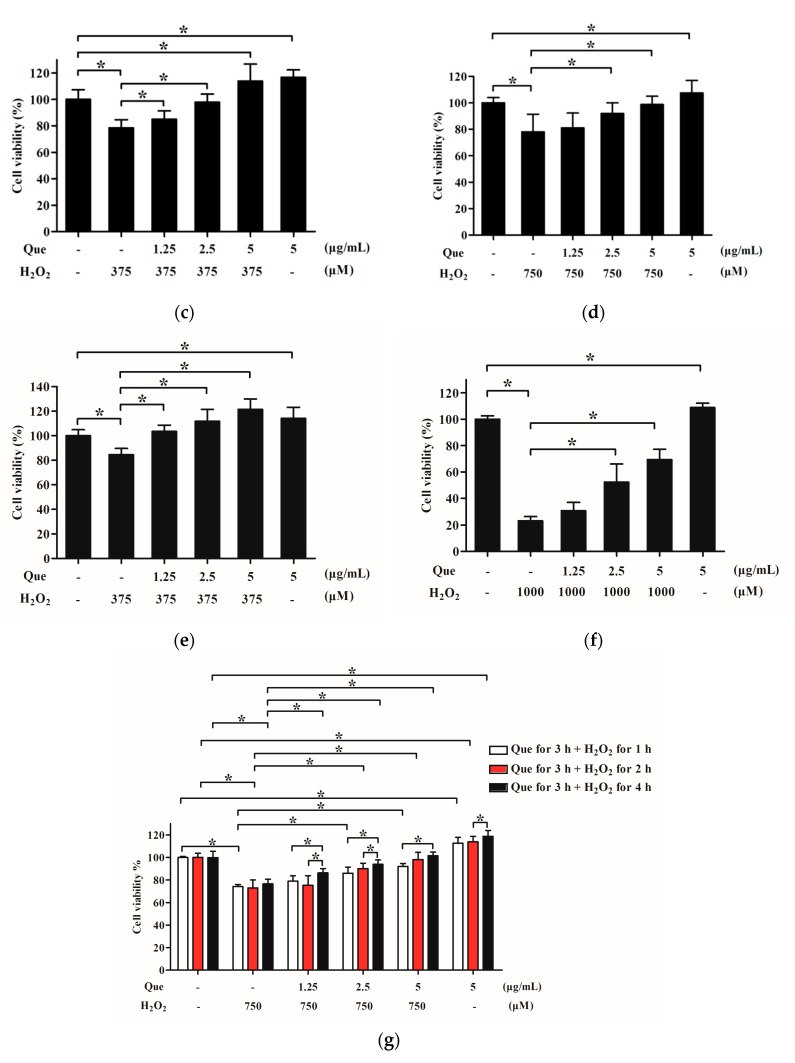

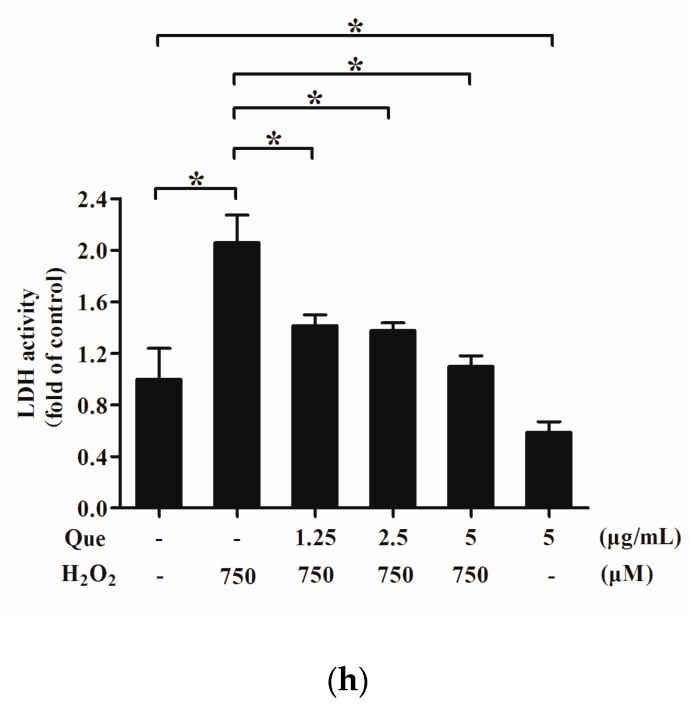
Effects of Que on damage of IPEC-J2 exposure to H_2_O_2_. (**a**) Effects of H_2_O_2_ on cell viability of IPEC-J2. After seeded for 24 h, cells were incubated with H_2_O_2_ for 24 h. (**b**) Effects of H_2_O_2_ on cell viability of IPEC-J2. After seeded for 24 h, cells were pre-incubated with DMEM for 20 h, then treated with DMEM containing H_2_O_2_ for 4 h. (**c**) Effects of pre-treatment with Que for 2 h then treatment with 375 μM H_2_O_2_ for 22 h on IPEC-J2 viability. (**d**) Effects of pre-treatment with Que for 20 h then treatment with 750 μM H_2_O_2_ for 4 h on IPEC-J2 viability. (**e**) Effects of co-treatment with Que and H_2_O_2_ for 24 h on IPEC-J2 viability. (**f**) Effects of pre-treatment with Que for 3 h then treatment with 1 000 μM H_2_O_2_ for 4 h on IPEC-J2 viability. (**g**) Effects of pre-treatment with Que with different concentration for 3 h then treatment with 750 μM H_2_O_2_ for 1 h, 2 h and 4 h on IPEC-J2 viability. (**h**) Effects of Que on LDH activity in culture medium of IPEC-J2 exposure to H_2_O_2_. IPEC-J2 were pre-treated with Que for 3 h then treated with 750 μM H_2_O_2_ for 4 h. Data are presented as mean ± SD. * *p* < 0.05 means significant difference between two groups.

For choosing appropriate concentrations of H_2_O_2_ with different treatment times to establish the oxidative damage model, we used the MTT assay method to determine the viability of IPEC-J2 exposed to H_2_O_2_. As shown in Figure 3a, treatment with at least 250 μM of H_2_O_2_ for 24 h resulted in a decrease of IPEC-J2 viability (*p* < 0.05). However, when cells were pre-cultured with DMEM for 20 h, then cultured with DMEM containing H_2_O_2_ ranging 750 μM to 2000 μM for 4 h, this caused a significant decrease in IPEC-J2 viability (*p* < 0.05) (Figure 3b). According to the results of Figure 3a,b, the H_2_O_2_ concentration of 375 μM, intermediate between 250 μM and 500 μM, was suitable to establish a long-term cell damage model as well as 750 μM and 1000 μM H_2_O_2_ were appropriate to obtain short-term cell damage models, respectively.

### 2.6. Effects of Que on Viability of IPEC-J2 Exposure to H_2_O_2_

In order to evaluate whether Que could relieve H_2_O_2_-induced cell damage, IPEC-J2 were treated with Que for different times, and thence or simultaneously challenged by H_2_O_2_ for diverse time. Cell viability was measured by the MTT assay method. IPEC-J2 viability was markedly decreased by H_2_O_2_ (*p* < 0.05) (375 μM for 22 h, as shown in Figure 3c; 750 μM for 4 h, as shown in Figure 3d; 375 μM for 24 h, as shown in Figure 3e; 1000 μM for 4 h, as shown in Figure 3f; 750 μM for 1 h, 2 h, and 4 h, as shown in Figure 3g). Short-time or long-time pre-incubation of Que (1.25–5 μg/mL) improved viability of IPEC-J2 Short-time or long-time exposure to H_2_O_2_, and co-incubation of Que and H_2_O_2_ for 24 h could also increase viability compared to H_2_O_2_ only-treated (*p* < 0.05) (Figure 3c–g). Interestingly, as illustrated in Figure 3g, as H_2_O_2_ treatment time increased, the viability-increasing effect of Que becomes more obvious in a roughly time and concentration- dependent manner. These results indicated that Que could protect IPEC-J2 from death.

### 2.7. Effects of Que on Lactate Dehydrogenase (LDH) Activity in Culture Medium of IPEC-J2 Exposure to H_2_O_2_

To further validate the protective effects of Que on IPEC-J2, we measured LDH activity in culture medium where IPEC-J2 were pre-treated with Que for 3 h then treated with 750 μM H_2_O_2_ for 4 h. As Figure 3h shows, H_2_O_2_ markedly raised LDH activity in IPEC-J2 culture medium, whereas Que reversed the increase of H_2_O_2_-mediated LDH activity in a concentration-dependent manner (*p* < 0.05). Interestingly, the LDH activity of Que-only treatment group is much lower than that of the control group (*p* < 0.05).

### 2.8. Effects of Que on Morphology of IPEC-J2 Cells Exposed to H_2_O_2_

**Figure 4:**
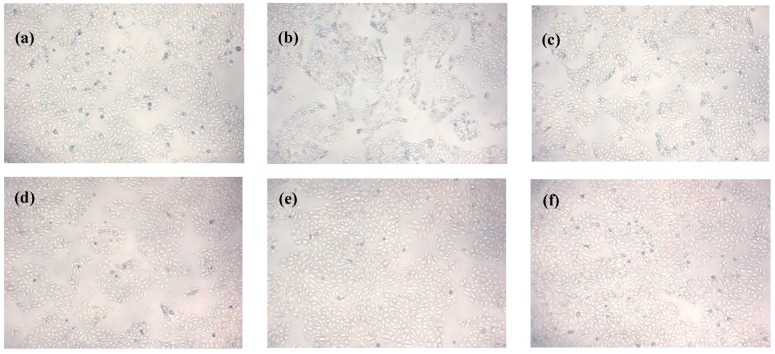
Effects of Que on morphology of IPEC-J2 exposure to H_2_O_2_. IPEC-J2 were pre-treated with Que for 3 h then treated with 750 μM H_2_O_2_ for 4 h. (**a**) 0 μg/mL Que + 0 μM H_2_O_2_; (**b**) 0 μg/mL Que + 750 μM H_2_O_2_; (**c**) 1.25 μg/mL Que + 750 μM H_2_O_2_; (**d**) 2.5 μg/mL Que + 750 μM H_2_O_2_; (**e**) 5 μg/mL Que + 750 μM H_2_O_2_; (**f**) 5 μg/mL Que + 0 μM H_2_O_2_. Observation multiple is 40×.

The morphology of IPEC-J2 exposed to Que (+/−) and H_2_O_2_ (+/−) was observed under an inverted microscope. As Figure 4a–f show, morphological changes were observed in IPEC-J2 exposed to 750 μM H_2_O_2_ for 4 h, such as shrunken cells, round shapes, and fewer normal cells, compared with the control group. After Que pre-treatment, cell numbers were increased and the morphology improved significantly, which were in a concentration dependent manner. Moreover, after 5 μg/mL Que pretreatment, the numbers and status of IPEC-J2 were basically similar to those of the control group. Besides, the number and state of cells treated with 5 μg/mL Que alone were markedly better than those in the control group.

### 2.9. Effects of Que on Apoptosis of IPEC-J2 Exposure to H_2_O_2_

**Figure 5:**
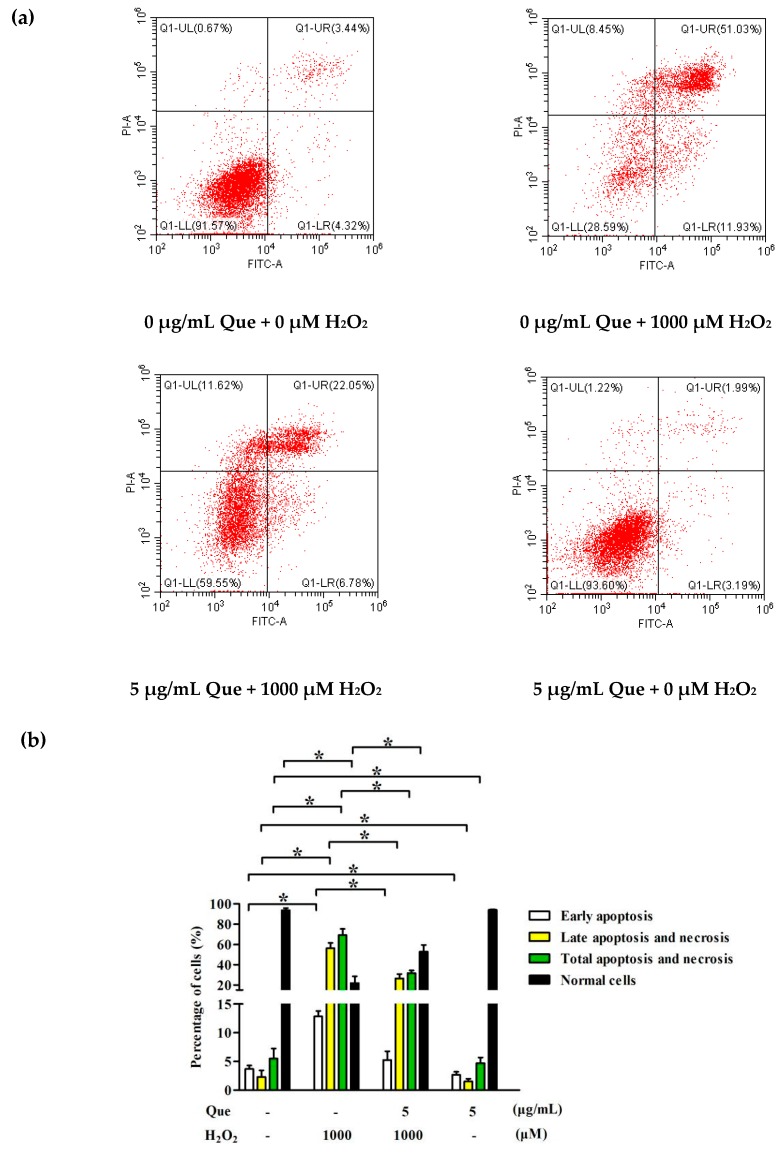

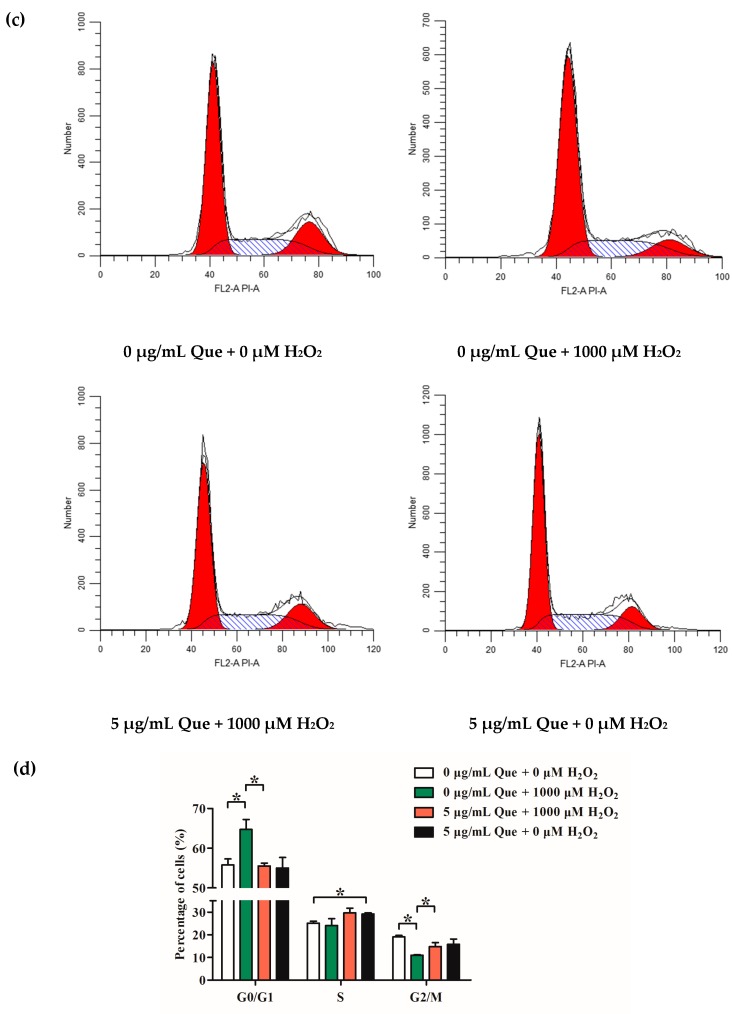
Effects of Que on apoptosis and cell cycle of IPEC-J2 exposure to H_2_O_2_. IPEC-J2 were pre-treated with Que for 3 h then treated with 1000 μM H_2_O_2_ for 4 h. (**a**) Flow detection diagram of apoptosis of IPEC-J2; (**b**) Histogram of apoptosis of IPEC-J2. (**c**) Flow detection diagram of cell cycle of IPEC-J2; (**d**) Histogram of cell cycle of IPEC-J2. Data are presented as mean ± SD. * *p* < 0.05 means significant difference between two groups.

To further elucidate whether the protective effect of Que was associated with a reduction of apoptosis, apoptosis was measured by Annexin V-FITC/PI staining. As Figure 5a,b show, H_2_O_2_ markedly increased the early apoptosis rate and late apoptosis and necrosis rate of IPEC-J2, as well as decreased the percentage of normal cells (*p* < 0.05). whereas Que reversed the apoptosis and necrosis rates changes and percentage of normal cells challenged by H_2_O_2_ (*p* < 0.05).

### 2.10. Effects of Que on Cell Cycle of IPEC-J2 Exposure to H_2_O_2_

To analyze the protective effect of Que on IPEC-J2, we further assayed the cell cycle distribution of IPEC-J2 dyed by PI via flow cytometry. As Figure 5c,d show, the percentage of G0/G1 phase cells was raised (*p* < 0.05), the percentage of S phase cells was not altered (*p* > 0.05), and the percentage of G2/M phase cells declined (*p* < 0.05), after H_2_O_2_ exposure, while 5 μg/mL Que reduced the percentage of G0/G1 phase cells, and promoted the percentage of S phase cells and G2/M phase cells of IPEC-J2, compared to the control group (*p* < 0.05).

### 2.11. Effects of Que on ROS Generation of IPEC-J2 Exposure to H_2_O_2_

**Figure 6:**
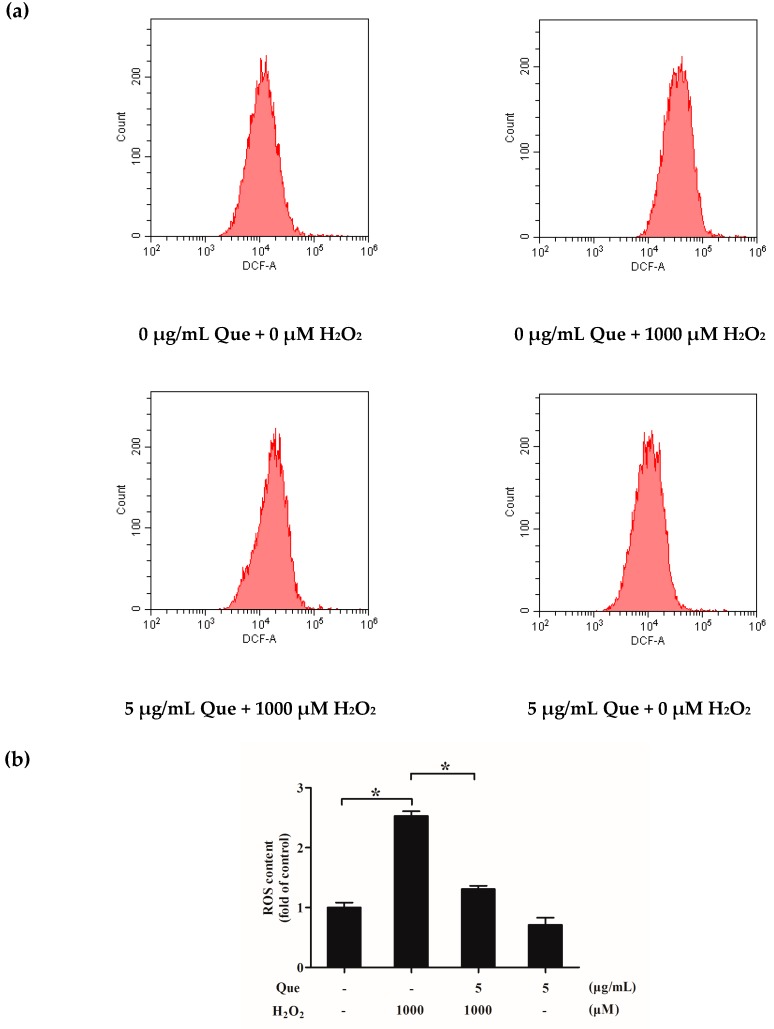

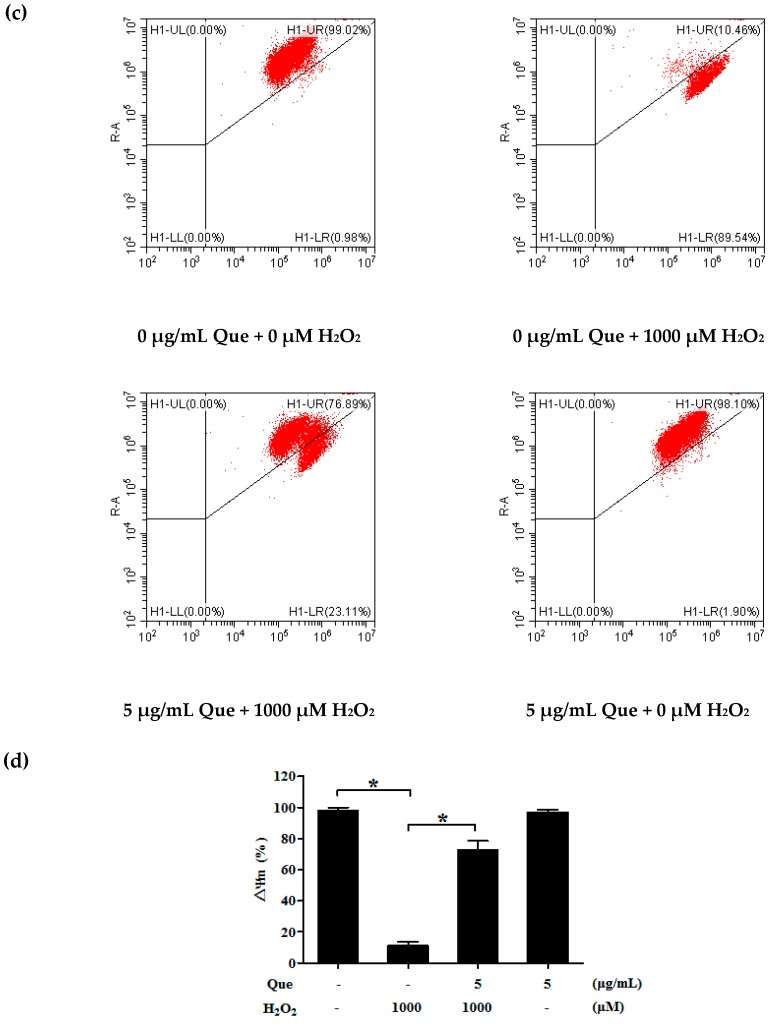
Effects of Que on ROS generation and Δψm of IPEC-J2 exposure to H_2_O_2_. (**a**) Flow detection diagram of ROS content of IPEC-J2; (**b**) Histogram of ROS content of IPEC-J2. (**c**) Flow detection diagram of Δψm of IPEC-J2; (**d**) Histogram of Δψm of IPEC-J2. Data are presented as mean ± SD. * *p* < 0.05 means significant difference between two groups.

To examine the protective effect of Que on IPEC-J2, we next determined ROS generation of IPEC-J2 dyed by 2′,7′-dichlorofluorescin diacetate (DCFH-DA). As Figure 6a,b show, the ROS content of H_2_O_2_-only treatment group was raised dramatically compared to the control group (*p* < 0.05). However, Que pre-incubation could suppress ROS generation after IPEC-J2 exposure to H_2_O_2_ (*p* < 0.05). Interestingly, Que-only incubation could also slightly reduce ROS generation, when compared to the control group, but there was no significant difference (*p* > 0.05).

### 2.12. Effects of Que on Mitochondrial Membrane Potential (Δψm) of IPEC-J2 Exposure to H_2_O_2_

To explore whether the protective effect of Que on IPEC-J2 was related to Δψm change, Δψm of IPEC-J2 was determined via flow cytometry after dying by 5,5′,6,6′-tetrachloro-1,1′,3,3′-tetraethyl- imidacarbocyanine iodide (JC-1). Figure 6c,d show that Δψm of IPEC-J2 was significantly reduced by H_2_O_2_ (*p* < 0.05). The obvious decrease of Δψm was reversed by Que at a concentration of 5 μg/mL, partly (*p* < 0.05).

### 2.13. Effects of Que on Bax/Bcl-2 of IPEC-J2 Exposure to H_2_O_2_

**Figure 7:**
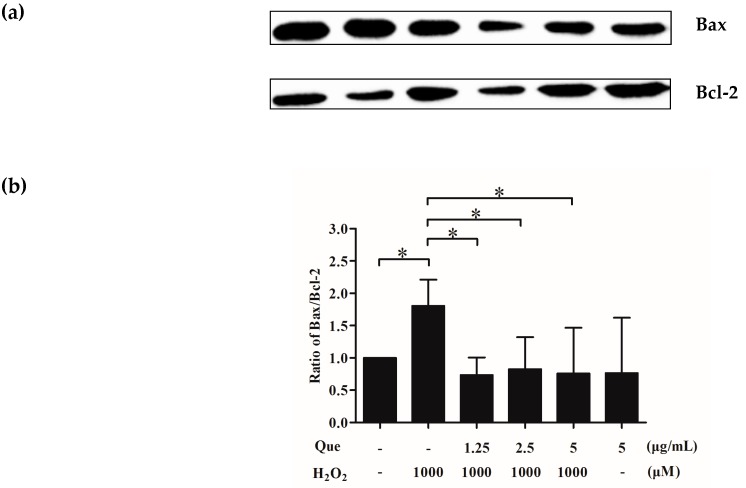
Effects of Que on Bax/Bcl-2 of IPEC-J2 exposure to H_2_O_2_. (**a**) Bands of Bax and Bcl-2 of IPEC-J2 by western blot; (**b**) Histogram of Bax/Bcl-2 of IPEC-J2. Data are presented as mean ± SD. * *p* < 0.05 means significant difference between two groups.

The ratio of Bax and Bcl-2 of IPEC-JE was evaluated by western blot. Figure 7a,b show that H_2_O_2_ obviously increased the ratio of Bax and Bcl-2, compared to control group (*p* < 0.05), whereas Que at 5 μg/mL reduced the H_2_O_2_-mediated increasing effect on value of Bax/Bcl-2, dramatically (*p* < 0.05).

### 2.14. Effects of Que on Lipid Peroxidation of IPEC-J2 under Low-Degree Oxidative Damage

**Figure 8:**
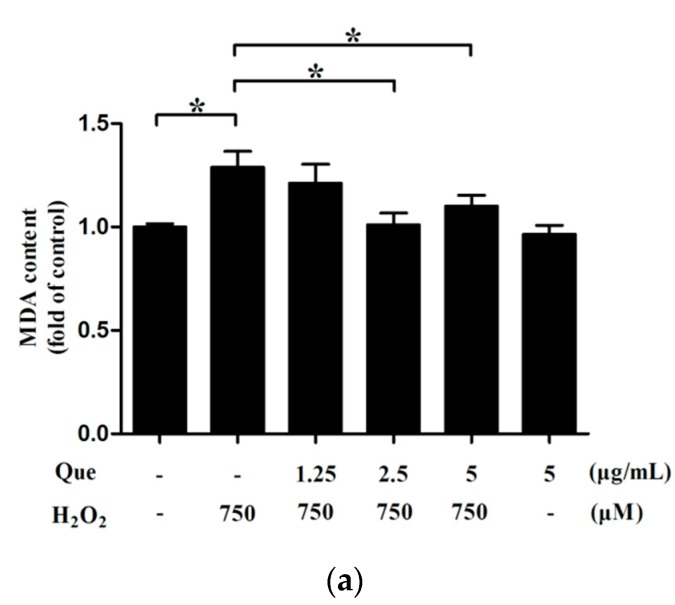

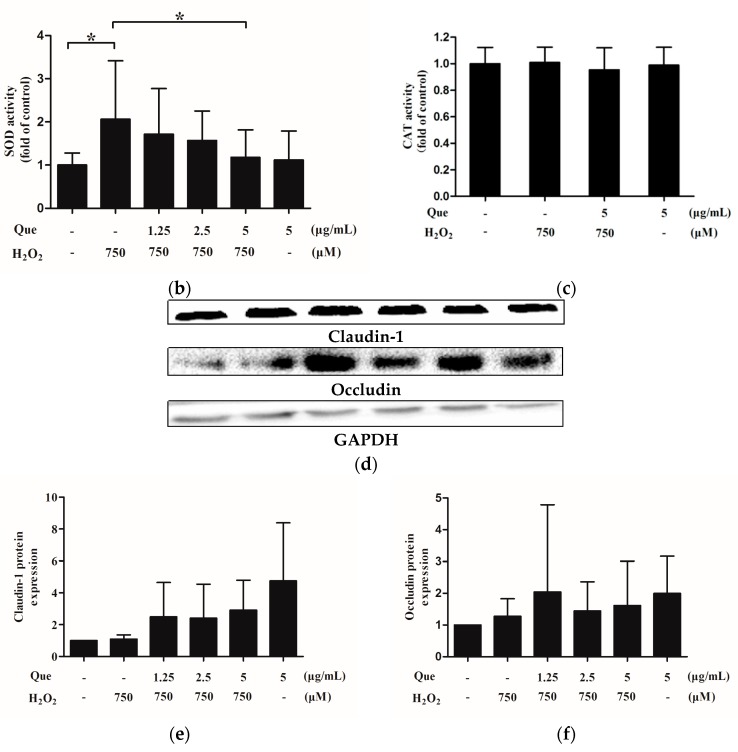
Effects of Que on redox system and tight junction proteins of IPEC-J2 under low-degree oxidave damage. IPEC-J2 were pre-treated with Que for 3 h then treated with 750 μM H_2_O_2_ for 2 h. (**a**) Effect of Que on MDA content of IPEC-J2. (**b**) Effect of Que on activity of SOD of IPEC-J2. (**c**) Effect of Que on activity of CAT of IPEC-J2. (**d**) Effects of Que on claudin-1 and occludin protein expression levels of IPEC-J2. Protein bands are presented as claudin-1 and occludin of IPEC-J2 by western blot. (**e**) Effect of Que on Claudin-1 expression level of IPEC-J2. The histogram shows relative gray values of claudin-1 of IPEC-J2. (**f**) Effect of Que on occludin expression level of IPEC-J2. the histogram shows relative gray values of occludin of IPEC-J2. Data are presented as mean ± SD. * *p* < 0.05 means significant difference between two groups.

To validate the effect of Que on lipid peroxidation of IPEC-J2 under low-degree oxidative damage, we measured malondialdehyde (MDA) content in IPEC-J2, where IPEC-J2 were pre-treated with Que for 3 h and then treated with 750 μM H_2_O_2_ for 2 h. The MDA content of IPEC-J2 was significantly increased in the low-degree oxidative damage group (*p* < 0.05). In addition, the MDA content of IPEC-J2 markedly declined after Que treatment, in a roughly concentration-dependent manner (*p* < 0.05) (Figure 8a).

### 2.15. Effects of Que on Activity of Superoxide Dismutase (SOD) and Catalase (CAT) of IPEC-J2 under Low-Degree Oxidative Damage Conditions

In order to determine the effect of Que on the antioxidant enzyme activity of IPEC-J2 under low-degree oxidative damage conditions, we assessed the activities of SOD and CAT. As shown in Figure 8b, SOD activity, induced by H_2_O_2_ was remarkably increased (*p* < 0.05). However, Que at 1.25, 2.5, and 5 μg/mL showed great inhibition in SOD activity, compared with the H_2_O_2_ treatment group (*p* < 0.05). In addition, the CAT activity of IPEC-J2 in the Que alone group and Que pretreatment group or H_2_O_2_ treatment group showed no differences with the control group (*p* > 0.05) (Figure 8c).

### 2.16. Effects of Que on Claudin-1 and Occludin Expression of IPEC-J2 under Low-Degree Oxidative Damage Conditions

To investigate the effect of Que on tight junction proteins of IPEC-J2 under low-degree oxidative damage conditions, expression levels of claudin-1 and occludin were assessed by western blot. As shown in Figure 8d,e, the claudin-1 expression level was unchanged after IPEC-J2 were challenged with 750 μM H_2_O_2_ for 2 h, compared with control group (*p* > 0.05). However, Que at 1.25, 2.5 and 5 μg/mL showed a slight promotion in claudin-1 expression level, compared with H_2_O_2_ treatment group (*p* > 0.05). Moreover, the occludin expression level of IPEC-J2 was also not changed in the H_2_O_2_ treatment group compared to the control group (*p* > 0.05), and it presented a mild increase in occludin expression level of IPEC-J2 after Que incubation, too (*p* > 0.05) (Figure 8d,f).

## 3. Discussion

In China, pig farming accounts for a large proportion of animal husbandry. The intestine of pigs is one of the important safeguards, and the intestinal health is a vital factor to ensure the normal development of the pig industry. The small intestine has the physiological functions of digestion, absorption and immunity. The proliferation of small intestinal epithelial cells has an effect on the intestinal villi and intestinal mucosa, which regulate the absorption and the immune function of the small intestine [14,15]. Li et al. [14] and Liu et al. [31] reported that *Macleaya cordata* alkaloids and *Rheum tanguticum* polysaccharides promoted the proliferation of IPEC-J2 and rat intestinal epithelial cells (IEC-6), respectively. In this study, we found that Que in low concentration increased IPEC-J2 viability in a short time, and its promoting effect was almost same as that in the serum treatment group. However, when the treatment time was longer, the drug showed a slower rate of increase in cell viability. We speculated that in contact with drugs for a long time, the cells would continue to uptake drugs, and intracellular drugs were hard to metabolize and utilize, so the concentration of intracellular drugs was higher and it then slowed down the proliferation rates of IPEC-J2. Basically similar to another study, after IPEC-J2 treated with chlorogenic acid for 1 h, the proportion of living cells in IPEC-J2 was slightly increased, but the proportion of living cells decreased significantly after incubation with the drug for 4 h and 24 h [32].

The cell cycle is closely related to cell proliferation, differentiation and death [33]. G1 phase is the DNA pre-synthesis stage, S phage is the DNA synthesis phase, and G2 phage is the DNA post-synthesis stage, which provide sufficient preparation for the subsequent division of cells [34,35]. Meanwhile, the ratio of the proportion of S phase and G2 phase cells to the proportion of G1 phase, S phase and G2 phase cells, were generally used as a cell proliferation index to reflect cell proliferation efficiency [36]. These results suggested that after incubation for 24 h with 5 μg/mL Que, IPEC-J2 were pushed into the S phase and the cell cycle in G1 phase was postponed. This indicated that Que might accelerate DNA synthesis to promote cell division. Meanwhile, our findings indicated that the cell proliferation index of Que treatment for 24 h was 124.13% of the control group, which was approximately in accordance with the results of cell vitality promotion measured by the MTT assay method. These data fully demonstrated that Que had great IPEC-J2 proliferation- promoting characteristics.

The cell cycle is regulated by cyclins, cyclin dependent kinases (CDKs), and cyclin dependent kinase inhibitors (CDKis) [37]. In the regulation process, cyclins and CDKs that correspond to cyclins, form complexes, while CDKis, such as P21 and P27, negatively regulated cell cycle by inhibiting the activity of cyclin-CDK complexes or CDK activity [38,39]. P21 has inhibitory effects on various CDKs activities, P27 inhibits CDK2/4 activities, to play a regulatory role in G1 phase and G1/S phase [39,40]. Our results demonstrated that the abundance of P21 and P27 genes decreased significantly after Que treatment. These results indirectly reflected that Que might decrease the inhibitory effects of CDKis on the activities of CDK2, CDK4 or CDK6, and CDK2/4/6-cyclin D1 complexes, or CDK2-cyclin E complex. Wu et al. [41] also demonstrated that the expression of P21 was decreased in the process of promoting proliferation of osteoblasts by serum of Jian Gu granules. Moreover, Qiu et al. [42] found that the expression of P21 and P27 was increased, which was linked with inhibitory proliferation of IPEC-J2 by sodium butyrate.

The cell scratch test is the most common research method in tumor cells, which is generally applied to evaluate the effect of drugs on tumor cell migration [43]. In addition, the effect of drugs on the repair of normal cells after mechanical injury can be evaluated by cell scratch test [44]. We found that Que did not promote repair of IPEC-J2 after scratching. Especially after 72 h, the high concentration of Que had a higher damage rate than that without Que treatment, which was shown as a certain effect of aggravation of damage.

During the process of breeding, poisoning of heavy metals, mycotoxins, and weaning stress, can all induce oxidative damage of porcine intestinal epithelial cells [20,21,22]. H_2_O_2_ is a common model substance for oxidative damage. Although some natural molecules from plants are antioxidants, but it does not mean that their antioxidant property is effectual to cells from all species [45,46]. Therefore, we explored whether the antioxidant Que could alleviate IPEC-J2 damage induced by H_2_O_2_. Our results showed that whether a long time or a short time preconditioning of Que, or simultaneous treatment of Que with H_2_O_2_, could play a good protective role in IPEC-J2, making cells free from or less damaged by H_2_O_2_. Interestingly, cell viability of 750 μM H_2_O_2_ exposure for 4 h was not significantly different from that of 750 μM H_2_O_2_ exposure to 1 h, 2 h. Nevertheless, 1.25 μg/mL to 5 μg/mL Que pretreatment for 3 h increased the vitality of cells exposed to 750 μM H_2_O_2_ for 4 h, whose degree of improvement was higher than that of cells exposed to 750 μM H_2_O_2_ for 1 h and 2 h. It was possible that Que continuously scavenged intracellular and external free radicals directly or indirectly and with the increase of time, more free radicals was eliminated, to reduce the amount of cell death.

Normally, LDH mainly exists in the cells. After the cells membrane is ruptured, a large amount of LDH is released to the extracellular medium [47]. The data in our study proved that LDH was seriously released from cells to the culture supernatant after H_2_O_2_ exposure, while the activity of LDH in the cell supernatant was decreased by Que pretreatment. This indicated that Que could resist H_2_O_2_ induced IPEC-J2 damage. Even in the Que control group, LDH activity in the supernatant was also significantly lower than that in the control group. We speculated that due to the lack of serum containing some nutrient substances, cells were damaged with starvation [48]. Que also had a protective effect on starving IPEC-J2. Simultaneously, these findings could also be verified by the morphological character of the cells treated with Que or/and H_2_O_2_. In the control group, the death cells were floating less in the supernatant. And some cells in only H_2_O_2_ treatment group died and many cells were crinkled. In Que pre-treating and only-treating group, the number of cell death was significantly reduced, and the state was also improved.

In the physiological state, there is a small amount of ROS in the animal body, which acts as a signal molecule to support normal physiological activities [49]. Once oxidative damage occurs, an excessive ROS can be produced, which is more likely to induce apoptosis [50]. Our results showed that the rates of early apoptotic cells as well as late apoptotic and necrotic cells were increased dramatically after cells were treated for 4 h with 1000 μM H_2_O_2_, which were reversed by 5 μg/mL Que. It indicated that Que possessed a good anti-apoptotic effect on IPEC-J2 co-incubated with H_2_O_2_. Apoptosis is often associated with cell cycle arrest [42]. The results suggested that H_2_O_2_ blocked the cells in the G0/G1 phase, and Que reduced the proportion of G0/G1 phase cells, increased the total proportion of S phase and G2/M phase cells, then promoted smooth entry of G0/G1 cells into S phase, to make the cell division and proliferation in order.

ROS mainly refers to H_2_O_2_, superoxide anion, hydroxyl radical, etc. [49]. H_2_O_2_ was cultured with cells, resulting in the increase of intracellular superoxide anion [51]. As a free radical, H_2_O_2_ can also probably enter cells and produce other free radicals inside the cells. Our results showed that H_2_O_2_ increased the intracellular ROS level significantly, but ROS content in IPEC-J2 cells was decreased dramatically by Que. Que might scavenge ROS through two ways. One is that Que directly acts on intracellular and external superoxide anion radical or other free radicals, then removes them [52,53,54]. The second way was that Que enters into cells to initiate the antioxidant pathway of cells, for promoting the production of antioxidant enzymes, and subsequently catalyzing and degrading ROS [55].

Under oxidative damage conditions, reduced mitochondrial membrane potential could influence mitochondrial integrity and energy release [21]. This would further interfere with the normal physiological function of cells [56]. We found that H_2_O_2_ significantly decreased Δψm, while Que pretreatment greatly increased the Δψm, reducing the mitochondrial damage caused by ROS. This indicated the apoptosis induced by H_2_O_2_ was related to the mitochondrial pathway, and the mechanism by which Que reduced apoptosis of IPEC-J2 was to inhibit the endogenous mitochondrial pathway.

Bax and Bcl-2, important proteins to regulate apoptosis, are important members of the Bcl-2 protein family, whose functions are to promote cell apoptosis and inhibit cell apoptosis, respectively [50]. Generally, the ratio of Bax to Bcl-2 is used to evaluate the degree of apoptosis. When the ratio increases, and apoptosis will be aggravated [57]. The results revealed that H_2_O_2_ increased the ratio of Bax to Bcl-2, resulting in exacerbating apoptosis of IPEC-J2, and Que reduced the ratio of Bax to Bcl-2 in IPEC-J2, showing a better inhibitory effect on apoptosis. Interestingly, 1.25 μg/mL of Que was enough to completely restore the ratio of H_2_O_2_ treatment back to that of untreated cells, which was not completely consistent with the results shown above of apoptosis, cell cycle progression, ROS generation and Δψm. The possible cause may be that other pathway(s) than the one mediate(s) Que-dependent prevention of H_2_O_2_-induced apoptosis.

Therefore, under conditions of oxidative damage by H_2_O_2_, excessive ROS stimulated cells, and then increased the value of Bax/Bcl-2, attacked the mitochondria to cause Δψm to decrease, might release the cytochrome c, and might be through caspase cascade reaction, eventually leading to apoptosis [56]. Que played a great inhibitory effect on H_2_O_2_-induced apoptosis in IPEC-J2 during the whole process, and its mechanism was related to the inhibition of endogenous mitochondrial pathway.

Most of the studies focused on the changes in the redox system of cells under the condition of more serious oxidative damage (cell vitality was about 50%) and under these severe oxidative damage conditions, some exogenous antioxidants increased the antioxidant enzyme activities decreased by oxidative stress [50,58]. However, during the initial stage of oxidative damage, there was a protective change in some antioxidant enzymes [59,60]. There was no definite information about whether the exogenous drugs pretreatment will reverse these changes in low-degree oxidative cells. SOD transforms superoxide anion into H_2_O_2_, and then CAT catalyzes H_2_O_2_ into water and oxygen, so as to eliminate ROS and protect cells from H_2_O_2_ damage [47]. Besides, MDA is the product of lipid peroxidation, and it is also one of the important indicators reflecting lipid damage [61]. In the present study, it was found that the MDA content was increased under the condition of 750 μM H_2_O_2_ incubation for 2 h, indicating that the cells were still damaged, which was associated with promoting lipid peroxidation in membrane. However, SOD activity was increased and CAT activity was not altered under the oxidative damage condition. We speculated that IPEC-J2 spontaneously mobilized enough SOD to play a protective role under mild oxidative injury, so it showed a high level in SOD activity. As CAT in IPEC-J2 was subjected to double stress of exogenous H_2_O_2_ and endogenous H_2_O_2_ transformed from other free radicals such as intracellular superoxide anion, CAT activity in H_2_O_2_ challenged group was not increased, but equivalent to the control group, which was not consistent with that of SOD activity. Que pretreatment reduced the degree of lipid peroxidation, and restore SOD activity to normal level, and maintain CAT activity in normal condition. In brief, treatment of Que reversed SOD activity and MDA content in H_2_O_2_-induced low-degree damaged cells. This might be related to the preferential removal of free radicals of Que.

Claudins and occludin are common tight junction proteins, which play a very important role in forming barriers and preventing the invasion of some exogenous pathogenic factors [62]. The decreased expression of claudin-1 and occludin is not conducive to maintaining intestinal integrity and affecting animal health. Interestingly, in present study, it was found that the relative expressions of claudin-1 and occludin were not significantly changed under the condition of low-degree oxidative damage of IPEC-J2, but the expression levels of occludin and claudin-1 of IPEC-J2 treated with Que showed a slightly upward trend, which indicated that Que might have the potential to up-regulate the expressions of claudin-1 and occludin. A previous study reported that after cells were treated with a lethal concentration of H_2_O_2_, the expression of claudin-1 gene in chicken primary intestinal epithelial cells was also not decreased [63]. Contrary to our results, ochratoxin A down-regulated the expression of tight junction protein occludin in duck intestines [64]. Moreover, claudin-1 and occludin in porcine gut were also affected by lipopolysaccharide, resulting in a decrease in the expression of these two proteins [65]. Thus, we inferred that no change in relative expressions of claudin-1 and occludin under low-degree oxidative damage in the present study might be associated with the time and concentration of reagent treatment. Moreover, the relative expression of claudin-1 and occludin would be affected by the types of substance tested.

## 4. Materials and Methods

### 4.1. Materials and Solution Preparation 

Dulbecco’s Modified Eagle Medium (DMEM), trypsin, penicillin-streptomycin, sterile phosphate buffered saline (PBS) and 3-(4,5-di-methylthiazol-2-yl)-2,5-diphenyltetrazolium bromide (MTT) were obtained from Procell Life Science Co., Ltd. (Wuhan, Hubei, China). Fetal bovine serum (FBS) was obtained from Hangzhou Sijiqing Bio-Engineering Material Co., Ltd. (Hangzhou, Zhejiang, China). H_2_O_2_ and Que were purchased from Sinopharm Chemical Reagent Co., Ltd. (Shanghai, China) and Shanghai Yuanye Bio-Technology Co., Ltd. (Shanghai, China), respectively. Sterile H_2_O_2_ stock solution (1 × 10^6^ μM) as well as Que stock solution (20 mg/mL) was prepared in PBS and dimethyl sulfoxide (DMSO), respectively. The final concentrations of H_2_O_2_ vehicle PBS and Que vehicle DMSO in fresh culture medium were no more than 5% and 0.2%. The H_2_O_2_ and Que stock solution were stored at −20 °C and used within 1 week and 3 weeks, respectively. Bax, Bcl-2, claudin-1, occludin, and glyceraldehyde phosphate dehydrogenase (GAPDH) antibodies were from Beijing Boiss Biotechnology Co., Ltd. (Beijing, China).

### 4.2. Cells Culture

IPEC-J2 were purchased from GuangZhou Jennio Biotech Co., Ltd. (Guangzhou, China). IPEC-J2 were cultured in DMEM supplemented with 10% FBS, 1% of penicillin (100 U/mL) and streptomycin (100 U/mL). IPEC-J2 were maintained in a humid atmosphere of 5% carbon dioxide and 95% air at 37 °C.

### 4.3. Cell Viability Assay

Cell viability was measured using the MTT assay method. For investigating the effect of Que on cell viability, IPEC-J2 were seeded at a density of 1.5 × 10^3^ cells/well in a 96-well plate and pre-cultured for 24 h. The cells were treated with 1.25 μg/mL to 40 μg/mL Que for 24 h and then with nontoxic concentration Que for 9 h, 24 h, and 48 h. To obtain proper modeling concentration of H_2_O_2_, IPEC-J2 were treated with 250 μM to 2000 μM H_2_O_2_ for 24 h and 4 h. For exploring the effects of Que on IPEC-J2 injury induced by H_2_O_2_, the trial designs were shown in Table 1. After the indicated trial treatments, 20 μL MTT solution was added to each well containing 100 μL serum-free medium. The cells were cultured for 4 h in the incubator at 37 °C. And 150 μL DMSO was added to each well after discarding the culture medium. After 10 min lightly shake, the absorbance was quantified on an Microplate Reader (Thermo Fisher Scientific Inc., Shanghai, China) at 490 nm. The calculation formula of cell viability is as follows:

Cell viability (%) = (OD_treatment group_ − OD_blank group_)/(OD_control group_ − OD_blank group_) × 100 (1)

### 4.4. Measurement of Cell Cycle

To study effects of Que alone treatment on cell cycle, IPEC-J2 were cultured in 6-well plates at a density of 6.5 × 10^4^ cells per well and pre-cultured for 24 h. Then cells were cultured with 5 μg/mL Que for 24 h. For elaborating effects of Que pre-treatment on cell cycle of IPEC-J2 exposure to H_2_O_2_, IPEC-J2 were cultured in 6-well plates at a density of 1.4 × 10^5^ cells per well and pre-incubated overnight. Cells were cultured with 5 μg/mL Que for 3 h, then treated with H_2_O_2_ for 4 h. 

After the procedure mentioned above, we collected cells, and washed them twice with ice-cold PBS. Then the cells were fixed via 70% ice-cold ethanol overnight. Subsequently, cells were washed by PBS again, and incubated with RNase at concentration of 50 μg/mL at 37 °C for 30 min. propidium iodide (PI) solution was added for co-culture with cells at 4 °C, for 30 min, without light. Cell cycles were analyzed by flow cytometers (ACEA, San Diego; Beckman, Brea, CA, USA).

### 4.5. Measurement of mRNA Relative Levels

To demonstrate the effects of Que alone treatment on the relative gene levels of P21 and P27, IPEC-J2 were seeded in 96-well plates at a density of 1.5 × 10^3^ cells per well then adaptively cultured for 24 h. Then cells were incubated with 5 μg/mL Que for 9 h. After indicated treatments, Total RNA was obtained via using Trizol reagent according to the manufacturer’s protocol. RNA quantity was evaluated at 260 nm and 280 nm, before total RNA was reverse transcribed into cDNA via 5 × TransScript^®^ All-in-One SuperMix and gDNA remover (TransGen Biotech, Beijing, China) under the condition of 42 °C for 15 min, 85 °C for 5 s and 4 °C forever. Then TransScript^®^ Tip Green qPCR Super Mix (TransGen Biotech) was applied to determine the genes expression levels, according to the product’s instruction. Conditions of pre-denaturation were of 94 °C for 30 s, and conditions of 40 cycles of reaction were of 94 °C for 5 s and 60 °C for 30 s. The primers of target gene, P21 and P27, and house-keeping gene GAPDH are showed in Table 2. And 2^−ΔΔCt^ was calculated to express relative expression levels of the P21 and P27.

### 4.6. Measurement of Cell Repair after Scratching

A cell repair test was conducted by cell scratching as described in previous study with some modification [44]. Briefly, IPEC-J2 were seeded in 6-well plates and pre-cultured overnight. Cells were scratched by 10 μL lance-gun head perpendicular to the bottom of 6-well plates. Culture medium was replaced as a fresh medium containing Que at 0, 1.25, 2.5 and 5 μg/mL continue treated with IPEC-J2 for 0 h, 24 h, 48 h and 72 h. We collected the image and measured the width of the scratch. The ratio of the scratched width of the Que treating group to the width of the control group was used as the damage rate.

### 4.7. Determination of LDH Activity

IPEC-J2 were cultured in 96-well plates and allowed to attach for 48 h. Before incubation with 750 μM H_2_O_2_ for 4 h, cells were incubated with Que for 3 h. The cells culture medium was collected. After centrifuged, LDH of supernatant was determined using a LDH assay kit (Solarbio Life Sciences, Beijing, China), at 450 nm using a microplate reader, according to the manufacturer’s protocol, to reflect LDH release of cells.

### 4.8. Observation of Cell Morphology

After treated with Que for 3 h, cells were incubated with 750 μM H_2_O_2_ for 4 h. The images of cells were collected by image acquisition system at the same location.

### 4.9. Apoptosis Analysis

IPEC-J2 (1.4 × 10^5^/well) were plated in six-well plates to attach overnight and treated with different concentrations of Que for 3 h. After drug treatment, cells were incubated with H_2_O_2_ for 4 h. Cell apoptosis was evaluated by flow cytometer with an Annexin V-Fluorescein isothiocyanate (FITC)/PI apoptosis detection kit (Keygene Biotech, Nanjing, Jiangsu, China) referring to the manufacturer’s protocol. In brief, cells were harvested by trypsin without ethylenediamine tetraacetic acid (EDTA), and washed by ice-cold PBS twice. Annexin V-FITC and PI were added to mix with IPEC-J2. Then IPEC-J2 were incubated for 30 min in the dark. Flow cytometry was used to analyze the cells.

### 4.10. ROS Assay

IPEC-J2 were plated in six-well plates for attaching overnight. Cells were treated with Que for 3 h, then incubated with H_2_O_2_ for 4 h. Intracellular ROS level was estimated by flow cytometer with a ROS detection kit (Beyotime Biotech, Shanghai, China) according to the product’s protocol. Briefly, IPEC-J2 were incubated with fluorescent probe DCFH-DA. The cells were analyzed via a flow cytometry.

### 4.11. Δψm Determination

IPEC-J2 were treated with different concentrations of Que for 3 h, then incubated with 1 000 μM H_2_O_2_ for 4 h. Δψm of IPEC-J2 was analyzed by flow cytometer with a Δψm assay kit (Beyotime Biotech) according to the product’s protocol. In Brief, IPEC-J2 were dyed with JC-1 and measured by a flow cytometer.

### 4.12. Proteins Relative Expression Levels Assay

After Que and/or H_2_O_2_ treatment, cells were collected and lysed by 150 µL radio- immmunoprecipitation assay (RIPA) lysis buffer. After centrifugation at 4 °C, the supernatant was collected for determination of the total protein concentration. Samples were separated on sodium dodecyl sulfate-polyacrylamide gels (SDS-PAGE), and transferred to polyvinylidene fluoride (PVDF) membranes. PVDF membranes were blocked, and incubated with Bax, Bcl-2, claudin-1, occludin, GAPDH primary antibodies in Tris buffered saline (TBS) overnight at 4 °C, then with a secondary antibody at room temperature for 1 h. The protein blots were imaged with an enhanced chemi-luminescence detection method. GAPDH was used as a housekeeping protein as reference for claudin-1, and occludin. The values of Bax/Bcl-2, and ratios of claudin-1, occludin, to GAPDH were used.

### 4.13. Determination of Level of Lipid Peroxidation (MDA), and Activities of SOD and CAT

IPEC-J2 were cultured with Que for 3 h, then treated with 750 μM H_2_O_2_ for 2 h. After drug treatment, MDA content, and activities of SOD and CAT were determined by a colorimetry method, making reference to protocols of the MDA content assay kits (Solarbio Life Sciences) and SOD activity assay kits and CAT activity assay kits (Comin Biotech, Suzhou, Jiangsu, China).

### 4.14. Statistical Analysis

All data are expressed as mean ± standard deviation (SD). Statistical difference among means were tested by one-way ANOVA and independent-sample *t* test. All data were analyzed with Statistical Product and Service Solutions (SPSS) 17.0 (IBM, Armonk, NY, USA). Values of *p* < 0.05 were considered a significant difference.

## 5. Conclusions

In conclusion, the present study demonstrates that Que promotes IPEC-J2 growth, which is associated with boosting the percentage of S phase cells as well as inhibiting gene expression of P27 and P21. In addition, Que protects IPEC-J2 from oxidative damage-mediated apoptosis and the mechanism is related to inhibition of the mitochondrial apoptosis pathway. Interestingly, Que suppresses lipid peroxidation and regulates SOD activity in low-degree damage cells. Besides, Que improves tight-junctional proteins relative levels, slightly. This finding exhibits great effects of Que on proliferation enhancement of IPEC-J2 and protection of IPEC-J2 challenged by oxidative stress, which presents potential use in porcine intestinal health care.

## Figures and Tables

**Table 1:** Trial designs of the effects of Que on viability of IPEC-J2 treated with H_2_O_2_.

Trial NO.	Trial Designs
Trial 1	IPEC-J2 were pre-cultured for 24 h. Cells were pre-incubated with Que for 2 h, then incubated with 375 μM H_2_O_2_ for 22 h.
Trial 2	IPEC-J2 were pre-cultured for 24 h. Cells were pre-incubated with Que for 20 h, then incubated with 750 μM H_2_O_2_ for 4 h.
Trial 3	IPEC-J2 were pre-cultured for 24 h. Cells were co-incubated with Que and 375 μM H_2_O_2_ for 24 h.
Trial 4	IPEC-J2 were pre-cultured for 48 h. Cells were pre-incubated with Que for 3 h, then incubated with 1000 μM H_2_O_2_ for 4 h.
Trial 5	IPEC-J2 were pre-cultured for 48 h. Cells were pre-incubated with Que for 3 h, then incubated with 750 μM H_2_O_2_ for 1 h.
Trial 6	IPEC-J2 were pre-cultured for 48 h. Cells were pre-incubated with Que for 3 h, then incubated with 375 μM H_2_O_2_ for 2 h.
Trial 7	IPEC-J2 were pre-cultured for 48 h. Cells were pre-incubated with Que for 3 h, then incubated with 375 μM H_2_O_2_ for 4 h.

**Table 2:** Primer sequences for PCR amplification.

Gene	Primer Sequences		Note
P21	ACCCCTTCCCCATACCC	TTCCAAACACCCATGAAACTG	[66]
P27	GTCCCTTTCAGTGAGAACCGATAC	TTGCTGCCACATAACGGAATCAT	[67]
GAPDH	GGGCATGAACCATGAGAAGT	TGTGGTCATGAGTCCTTCCA	NM_001206359.1

## Supplementary Materials

The following is available online. Figure S1: Effects of Que on repair of IPEC-J2.
