# Supplementary material for: Effects of Quercetin on Proliferation and H2O2-Induced Apoptosis of Intestinal Porcine Enterocyte Cells
Source: Molecules. 2018 Aug 12;23(8):2012. doi: 10.3390/molecules23082012 (PMC6222514; doi:10.3390/molecules23082012)
Supplement: Supplementary file 1 [file molecules-23-02012-s001.pdf]

Supplementary Material for

Effects of Quercetin on Proliferation and H_2O_2-Induced Apoptosis of Intestinal Porcine Enterocyte Cells

Zhigang Chen [1], Qiaoling Yuan [1], Guangren Xu [1], Huiyu Chen [2], Hongyu Lei [2] and Jianming Su [1,*]

[1] Department of Basic Veterinary Medicine, College of Veterinary Medicine, Hunan Agricultural University, Changsha 410128, Hunan, China; czgresearcher@163.com (Z.C.); yql0501@126.com (Q.Y.); xugr0818@163.com (G.X.)

[2] Department of Preventive Veterinary Medicine, College of Veterinary Medicine, Hunan Agricultural University, Changsha 410128, Hunan, China; chenhuiyuhn@163.com (H.C.); leihy77@hunau.edu.cn (H.L.)

* Correspondence: sjmauhn@hunau.edu.cn (J.S.); Tel.: +86-731-8467-3860 (J.S.)

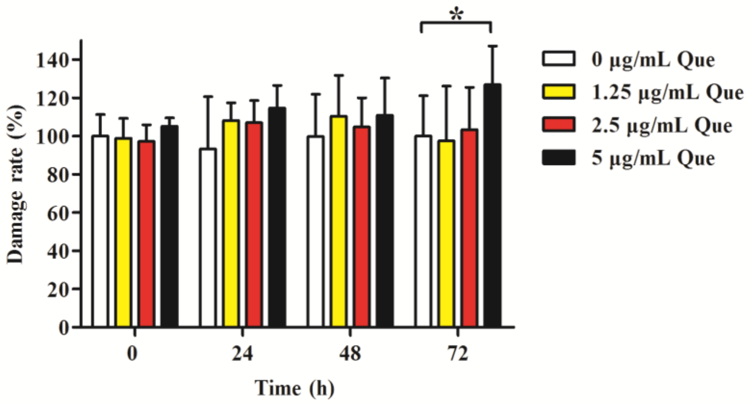

Figure S1. Effects of Que on repair of IPEC-J2. Data are presented as mean ± SD. * $p < 0.05$ means significant difference between two groups.
